# Immunohistochemical Assessment of Acute Myocardial Infarction: A Systematic Review

**DOI:** 10.3390/ijms26188901

**Published:** 2025-09-12

**Authors:** Gianpiero D’Antonio, Nicola Di Fazio, Lavinia Pellegrini, Alessandro Ghamlouch, Fabio Del Duca, Raffaele La Russa, Paola Frati, Aniello Maiese, Gianpietro Volonnino

**Affiliations:** 1 Department of Anatomical, Histological, Forensic and Orthopedic Sciences, Sapienza University of Rome, Viale Regina Elena 336, 00161 Rome, Italy; gianpiero.dantonio@uniroma1.it (G.D.); lavinia.pellegrini@uniroma1.it (L.P.); alessandro.ghamlouch@uniroma1.it (A.G.); paola.frati@uniroma1.it (P.F.); gianpietro.volonnino@unicamillus.org (G.V.); 2 Department of Life Sciences, Health and Health Professions, Link Campus University, 00165 Rome, Italy; n.difazio@unilink.it; 3 Department of Biomedicine and Prevention, University of Rome “Tor Vergata”, Via Montpellier 1, 00133 Rome, Italy; fabio.delduca@uniroma1.it; 4 Department of Clinical Medicine, Public Health, Life Sciences, and Environmental Sciences, University of L’Aquila, 67100 L’Aquila, Italy; raffaele.larussa@univaq.it

**Keywords:** acute myocardial infarction, immunohistochemistry, forensic pathology, cardiac markers, troponin, C5b-9, dystrophin, H-FABP, early post-mortem diagnosis, sudden cardiac death

## Abstract

In forensic medicine, spotting signs of an acute myocardial infarction (AMI) right after it happens is still a tough call, especially in sudden-death cases. Standard histology often misses changes in those critical first hours because the tissue damage is too subtle to see. To tackle this, we reviewed research (1990–2023) from PubMed and Web of Science, following PRISMA guidelines. We focused on studies that used immunohistochemistry to identify markers of early AMI in both human autopsies and animal models, specifically in the first six hours post-event. Our selection process narrowed 418 records to 37 key papers. We screened 49 markers in total, but only a handful stood out for reliable diagnosis: C5b-9, cardiac troponins, dystrophin, and H-FABP—all showing high specificity. Markers like S100A1 and IL-15 also showed promise, whereas JunB and connexin-43 appeared less dependable. We believe immunohistochemistry can add real value in early AMI identification, especially when using combinations of markers chosen for complementary strengths. Still, to make this approach practical in forensic settings, we need more studies on human samples and agreement on standardized lab protocols.

## 1. Introduction

Acute myocardial infarction (AMI) is one of the leading causes of sudden cardiac death worldwide, with a significant impact on public health [1]. According to the World Health Organization (WHO), ischemic heart disease was the leading cause of death in 2020, accounting for 16% of all deaths [2]. The incidence of myocardial infarction varies based on demographic and socioeconomic factors, affecting men more frequently than women, although the gap narrows with advancing age. The main pathophysiological mechanisms underlying ischemic heart disease include reduced coronary blood flow, decreased perfusion pressure gradient, narrowing of the coronary lumen, reduced oxygen availability in the circulating blood, and shortened perfusion time. A sudden reduction in coronary blood flow leads to decreased oxygen supply, causing cardiac ischemia and subsequent cell death [3].

From a morphological perspective, three distinct phases occur during the infarction process: ischemic necrosis, the inflammatory phase, and the repair phase. Following infarction, cell death activates specific inflammatory signaling pathways. Pro-inflammatory cytokines released in response to tissue damage promote neutrophil recruitment to the injury site, contributing to the inflammatory process and subsequent myocardial tissue repair.

Following a chronological order, it is possible to identify the timing of the infarction on the basis of the anatomo-pathological findings detected.

Within the first 4–6 h of ischemic onset, no significant macroscopic or microscopic tissue changes are observed. After 6 h, the first histological sign appears, characterized by thin, wavy fibers (waving) due to the absence of post-infarction contraction. However, these findings are neither highly sensitive nor specific. Around 24 h post-infarction, the affected area appears pale and soft macroscopically, while microscopically, the initial phase of coagulative necrosis becomes evident, marked by cellular swelling, pyknotic nuclei, hypereosinophilic sarcoplasm, loss of striations in myofibrils, and contraction band necrosis (also known as coagulative myocytolysis) [4]. By days 3–5, the infarcted area becomes sharply demarcated, and diffuse neutrophilic infiltration is observed, first in the intravascular site (margination) and subsequently in the interstitial space, signaling the onset of the inflammatory response. After 7–10 days, the necrotic process continues with the destruction of damaged muscle fibers and the accumulation of cellular debris, which are phagocytized by macrophages gradually replacing the neutrophilic infiltrate. During the next 2–3 weeks, fibrosis becomes more and more extensive, and scar tissue develops, replacing damaged muscle tissue. Residual myocardium no longer shows signs of acute necrosis and cardiac tissue repair is consolidated, leading to structural stabilization of the previously damaged area [5]. After approximately 3 months, histological dating of the ischemic event is no longer possible due to scar stabilization.

Despite the lack of universal agreement on the definition of sudden cardiac death (the WHO defines it either within 1 h of symptom onset if witnessed, or within 24 h of having been observed alive and symptom free if unwitnessed, while the Association for European Cardiovascular Pathology defines it as death within 6 h of symptom onset) [6,7,8,9], post-mortem diagnosis of myocardial infarction in the early phase (<4–6 h) remains a complex challenge in forensic practice [7,10].

Given the rapidity of this process, biochemical markers useful for diagnostic purposes must exhibit specific characteristics, reflecting myocardial cell structural alterations in response to acute hypoxia. Recently, research has focused on the use of microRNAs and immunohistochemistry—a relatively inexpensive and accessible technique—for AMI diagnosis and other forensic applications [11], offering significant potential to improve diagnostic accuracy. Immunohistochemistry, by exploiting the specific binding between antibody and antigen allows for the detection of cellular mediators before the appearance of histological inflammatory infiltrates [12,13,14].

The aim of our review is to analyze various immunohistochemical markers for early-phase acute myocardial infarction reported in the literature, identifying the most reliable and useful markers in forensic practice.

## 2. Methods

**Figure 1:**
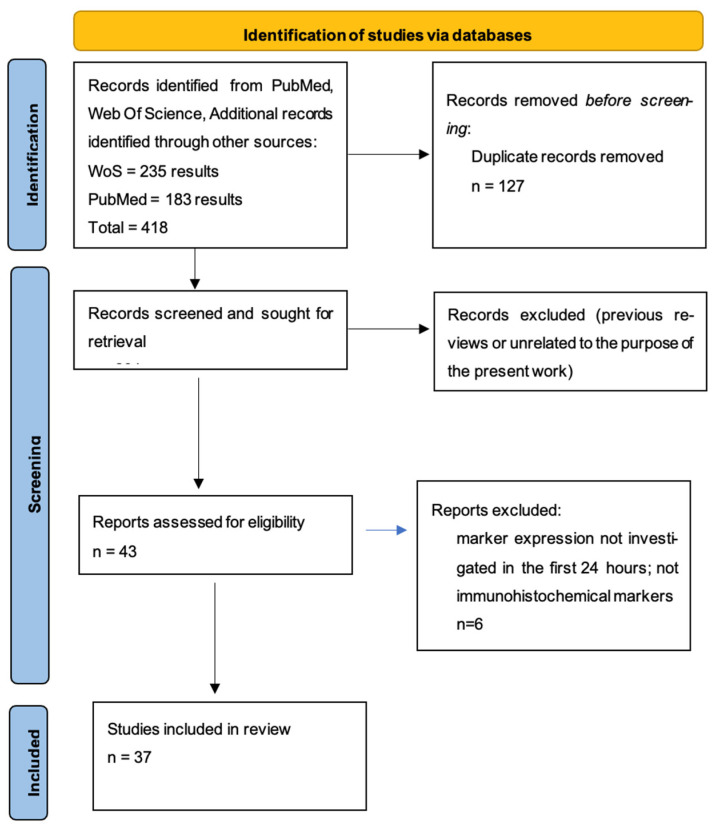
PRISMA flowchart.

The methodology and reporting of this systematic review follow the PRISMA (Preferred Reporting Items for Systematic Reviews and Meta-Analyses) guidelines to ensure transparency and reproducibility. A systematic literature review was conducted according to PRISMA guidelines using the PubMed and Web of Science databases as showed in Figure 1. The search strategy included the strings “immunohistochemical marker*” (All Fields) AND “myocardial infarction” (All Fields) for WoS, and “ALL = (Immunohistochemical marker*) AND ALL = (myocardial infarction)” for PubMed, selecting articles published between 1990 and 2023 [15]. Only articles in English were considered, obtaining an initial total of 235 results on Web of Science and 183 on PubMed, for a total of 418 papers.

After removing duplicates, 291 articles remained. Abstracts were screened, and in cases of uncertain relevance, the full text was evaluated. Review articles and studies not related to the topic were excluded, resulting in a final selection of 37 studies included in the review.

For each study, data regarding the presence or absence of marker expression in infarcted and non-infarcted myocardial tissue were systematically extracted. When numerical information was available on control groups, diagnostic specificity was calculated as the proportion of true negatives. When only descriptive data were available, specificity was estimated based on the number of control samples reported as negative or positive. In the absence of any usable data, specificity was not estimated.

## 3. Results and Discussion

The literature analysis highlighted numerous immunohistochemical markers used for the early detection of myocardial infarction in both animal and human models. The research yielded 37 scientific articles deemed suitable for analysis. Across these studies, 49 immunohistochemical markers were identified and examined to diagnose and determine the timing of early myocardial infarction (<4–6 h). Table 1 provides a detailed summary of the research articles included in the review. Several immunohistochemical markers proved to be particularly reliable, in terms of both sensitivity and specificity, for diagnosing acute myocardial infarction in its early phase. Fibronectin, C5-b9, Troponins and dystrophin are the most studied markers and can be considered the most reliable as well.

*Adropin*—Aydin et al. [16] studied adropin as a marker in a rat model, detecting positive expression as early as 1 h after the ischemic event, with a progressive increase up to 24 h, aligning these findings with the corresponding histological results.

*S100-A1* is a calcium-binding protein. It is primarily expressed at the cardiac level, in ventricular muscle cells, so a downregulation is associated with myocardial contraction dysfunction. Regarding immunohistochemistry, this marker has been studied in autopsy and animal samples. In cases of early myocardial ischemia, protein depletion has been observed. Bi et al. (2013) [17] analyzed S100A1 in both rats and human samples, observing early protein depletion as soon as 15 min in rats, with progression up to 6 h. In human samples with histological signs of myocardial infarction, massive depletion of S100A1 was observed. In humans with suspected MI but without histological signs, S100A1 depletion began 30 min after symptom onset and progressively intensified (45 min, 1 h, 2 h, 2.5 h, 4 h). In the control group (10 individuals who died from other causes), no S100A1 depletion was observed.

*Fibronectin* is a high-molecular-weight glycoprotein found in plasma and the extracellular matrix, playing a role in the cytoskeletal system, tissue repair, phagocytosis, and cell adhesion. Following the rupture of the plasma membrane due to ischemic injury, it can be detected inside the cell after approximately one hour. It is the most studied marker and proved to have good sensibility and specificity. Hu et al. [27,28] in a study on rats, reported fibronectin deposition in ischemic cardiomyocytes as early as 15–30 min after symptom onset. This finding contrasts with the data from Campobasso et al. [21], who observed significant expression only in cases with prolonged survival (8–10 h after symptom onset). In two studies conducted by Mondello et al. [40,41] a few years apart, fibronectin positivity was absent in all control cases of asphyxial death.

*Fibrinogen* is an acute-phase protein and a coagulation factor that plays a crucial role in thrombotic formation. In animal models studied by Hu et al. [27,28], positive expression was observed after 3 h. According to Brinkman et al. [19], fibrinogen expression in human samples occurred before 6 h after the ischemic event, with greater intensity compared to 4–9, which was studied simultaneously.

*C5b-9*—It forms the membrane attack complex (MAC) during the activation of the complement cascade. Once assembled on the membrane, it induces cell lysis. Brinkmann et al. [19], in a study on human samples, observed the early manifestation of C5b-9 in all cases with macroscopic and microscopic signs of infarction. In fact, it can be considered the gold standard among early expression markers, with the highest specificity. Some authors report its detection as early as 40 min after the event. However, according to Campobasso et al. [20,21], C5b-9 detection appears to be slower than the depletion of cardiac antigens such as troponin and myoglobin. In their study on rats, Sabatasso et al. [46,47] documented an increase in C5b-9 within 2 h of the ischemic event. Mondello et al. [40,41], comparing three autopsy groups, described clear positivity in samples with a confirmed infarction diagnosis, weak positivity in suspected MI cases, and negativity in control cases.

*Myoglobin* is an oxygen-binding protein found in the sarcoplasm of skeletal muscle fibers and the cytoplasm of cardiac cells. When the myocardium undergoes ischemic injury leading to cellular membrane damage, myoglobin depletion can be observed in both autopsy and animal samples. Myoglobin is an early marker of myocardial infarction, detectable as soon as 1–2 h after the onset of necrosis. Some studies on rats have reported immunohistochemical alterations as early as 15 min post-injury.

Although considered a sensitive and specific marker, Ortmann et al. [42] found myoglobin depletion in both confirmed and suspected cases of ischemic cardiac death. Similarly, Sabatasso et al. (47,48) observed variable myoglobin depletion across all study groups, including cases with macro- and microscopic evidence of infarction, unconfirmed infarction cases, and asphyxia-related deaths.

*Troponin* is a crucial protein complex involved in the regulation of muscle contraction. It consists of three main subunits: troponin C, which binds calcium and initiates contraction; troponin I, which inhibits actin-myosin interaction when the muscle is at rest; and troponin T, which binds to tropomyosin, stabilizing the complex. Present in both skeletal and cardiac muscles, troponin regulates contraction in response to calcium concentration changes.

In post-mortem samples, troponin depletion in cardiomyocytes is considered a reliable marker of myocardial ischemia. A study by Sabatasso et al. [46,47] documented troponin T and I depletion as early as 1 h after the ischemic event. Similarly, Jia et al., in a study on rabbits, observed an increase in cardiac troponin I (cTnI) expression 30 min after coronary occlusion, followed by its depletion after approximately 1 h. When analyzing a sample of 30 infarct-related deaths, they detected troponin I depletion in the infarcted area.

Although troponin I is often regarded as highly sensitive and specific, Martinez Diaz et al. [18], in a study of 50 autopsy samples, found that troponin T expression was more intense and frequent.

*H-FABP* (Heart-type Fatty Acid Binding Protein) is a fatty acid-binding protein found in the cytoplasm of myocardial cells. Due to ischemic damage, the protein leaks through the damaged cell membrane and enters the bloodstream within 30 min to 2 h after the onset of ischemia. Some authors have demonstrated that H-FABP is more sensitive than myoglobin for diagnosing myocardial infarction within the first 12 h of symptom onset. Meng et al. [39] conducted an immunohistochemical analysis on both autopsy and animal samples. Their study examined 36 mice, divided into six groups: a control group and five myocardial ischemia groups, evaluated at 15, 30 min, 1, 2, and 4 h post-ischemia. In mice, H-FABP positivity appeared as early as 15 min and progressively increased with ischemic duration. In human hearts, including cases of infarction with macroscopic and microscopic evidence, infarcts without microscopic confirmation, and control cases (death within 6 h of symptom onset), the results showed complete depletion of H-FABP in confirmed infarction cases, early positivity in suspected cases, with H-FABP depletion even in areas negative for myocardial ischemia under hematoxylin-eosin staining and normal expression in control cases. Similar results were reported by Shabaiek et al. [49], who conducted a study on autopsy-derived human heart samples.

*GDF-15/ET-1*—GDF-15 (Growth Differentiation Factor 15) is a cytokine belonging to the TGF-β (Transforming Growth Factor-β) family, involved in oxidative stress response, inflammation, and apoptosis. Endothelin-1 (ET-1) is a potent vasoconstrictor that regulates blood pressure and vascular tone. During myocardial infarction (MI), GDF-15 is released by cardiac cells, and ET-1 levels increase significantly.

Falk et al. [23] analyzed 29 rats using the Langendorff model. Among them, 24 rats underwent coronary ligation (ischemia without reperfusion), 5 experienced infarction followed by reperfusion, and 7 served as controls. In rats without reperfusion, both ET-1 and GDF-15 were positive within the first hour, in some cases as early as 5 min. The biomarkers were also detected in reperfused rats, while control group results varied. However, in formalin-fixed hearts, neither biomarker was detected, suggesting that fixation methods may influence detection outcomes. The same study also examined 41 human hearts, including 32 cases of suspected acute myocardial infarction based on histological criteria and clinical data, and 11 from individuals who had died from other causes. In hearts without histological alterations, GDF-15 was positive in 5 out of 6 cases, whereas ET-1 was detected in only one case. Among the 26 infarct cases, 25 showed positivity for GDF-15, and 17 also for ET-1. In the control group, GDF-15 was consistently positive, while ET-1 was present in 6 out of 9 cases with variable intensity. GDF-15 is a highly sensitive but poorly specific marker, whereas ET-1 is less sensitive but more selective for confirmed ischemic cases. However, neither proved to be a reliable indicator of early-stage acute myocardial infarction.

*Dystrophin* is a crucial protein for the stability and functionality of muscle cells, particularly skeletal and cardiac muscle fibers. It is part of the dystrophin–glycoprotein complex, linking the intracellular cytoskeleton (actin) to the extracellular matrix through the plasma membrane. Following myocardial infarction, dystrophin dissociates from the membrane. Immunohistochemical studies conducted by Mondello et al. [40,41] on human hearts observed dystrophin depletion in both confirmed and suspected infarct cases, with lower intensity in suspected cases, and negativity in controls. Hashmi et al. [25] analyzed dystrophin expression in 30 mice divided into six groups. In samples collected after 20 min, 30 min, and 1 h, no histological alterations were detected. After 4 h, histology remained negative except for some areas of eosinophilia. After 24 h, positive histological signs appeared. The control group showed no alterations.

*Fas-FasLl- P53-BCl-2-Bax*—Proteins that regulate programmed cell death can increase their expression in response to ischemic stress. According to the study by Piro et al. [45], which analyzed 16 cases of myocardial infarction with symptom onset between 3 and 12 h before death (some showing histological signs of ischemia) and 5 control cases, the reactions were consistently negative.

*Dityrosine* forms as a result of a reaction between tyrosyl radicals following oxidative stress. Mayer et al. [37,38] studied dityrosine in 29 rats with induced myocardial ischemia and 72 human autopsy samples. In the animal model, they analyzed 24 infarct cases with durations ranging from 5 to 60 min, 5 cases of infarction followed by reperfusion, and 7 controls. Dityrosine was positive in 22 of the 24 infarct cases and in all subjects who underwent reperfusion, whereas in the control group, it was detected only in the subepicardial region. In the human cohort, 72 subjects were analyzed, including 61 cases of cardiac-related death, 13 of which had no histological signs of ischemia within the first 0–4 h, and 11 controls. Dityrosine was detected from 4 h onward, but its low specificity was evident due to its expression in some control cases as well.

Some markers are associated with the inflammatory response and inflammation mediators, including *IL-1β*, *IL-6*, *IL-8*, *IL-15*, *MCP-1*, *TNF-α*, and *ICAM-1*. These cytokines and adhesion proteins indicate the activation of the inflammatory cascade in the context of acute myocardial infarction. Leukocyte infiltration in necrotic areas is often accompanied by the presence of these molecules.

IL-15, Monocyte Chemoattractant Protein-1 (MCP-1), IL-8, CD15, CD18, ICAM-1, TNF-α, and Tryptase regulate inflammation and immune response, working together to recruit immune cells and trigger inflammation following tissue damage, such as myocardial infarction. In a study conducted on human autopsy samples (26 infarct cases and 25 controls), Turillazzi et al. [50] observed a rapid and intense immunohistochemical expression of IL-15 and MCP-1 within 0 to 4 h. In contrast, CD15, tryptase, TNF-α, IL-8, CD18, and ICAM-1 showed lower expression levels.

*Jun-B* belongs to the AP-1 (Activator Protein-1) family, playing a role in cell differentiation, apoptosis, and proliferation. Sabatasso et al. [46,47], in a study on animal models, observed Jun-B expression approximately 30 min after occlusion of the left anterior descending coronary artery. In a subsequent study conducted in 2018, the same authors found that in myocardial ischemia cases, the alteration was mainly observed at the subendocardial level, whereas in asphyxia cases, Jun-B expression was higher, indicating low specificity of the marker.

*Cx43/np-Cx43*: Cx43 is a gap junction protein, known as connexin, located at the level of intercalated discs. Following an ischemic stimulus, the protein undergoes dephosphorylation (np-Cx43), repositioning within the cytoplasm and at the “edges” of cardiomyocytes. Studies on animal models, conducted on rats subjected to left anterior descending artery ligation, showed a progressively increasing expression of Cx43 in gap junctions within the ischemic region as early as 15 min after the ischemic insult. In a 2013 study, Kawamoto et al. [29] analyzed the np-Cx43 marker alongside Cx43 in 57 cases of sudden cardiac death and 24 control cases (deaths due to asphyxia). They observed a rapid alteration with Cx43 depletion and positive staining for np-Cx43 in confirmed acute myocardial infarction cases. Moreover, connexin 43 (Cx43) and np-Cx43 were also positive in cases without macroscopic or microscopic signs of infarction, suggesting the potential utility of the marker even in the absence of evident myocardial damage.

*Desmin*, *Vinculin*, *α-Actin*—Cytoskeletal and cellular architecture proteins. Their depletion or modification is an indicator of cellular damage, as cytoskeletal disruption is one of the hallmark events of myocardial necrosis. Zhang et al. [52], through a study on autopsy samples, observed immunohistochemical depletion of α-actin and vinculin within 1 h of ischemic injury. Regarding desmin expression, several authors reported a positive reaction (protein depletion) even in cases of early myocardial infarction without correlated histological signs. In Hu et al.’s study on rats, desmin and HHF35 actin depletion occurred as early as 15 min in the ischemia group, but not in the control group, suggesting the potential use of this marker even in early myocardial infarction cases.

*C9*: Piercecchi-Marti et al. [44] studied the expression of C9, a complement protein that plays a crucial role in the inflammatory response by forming the membrane attack complex, demonstrating a positive reaction in individuals who died one hour after the onset of typical myocardial ischemia symptoms, but in the absence of histological signs at autopsy.

The results of the study confirm the limitations of traditional morphology (H&E staining) in detecting very early myocardial ischemic damage. All the previously listed markers are, in fact, detected at an early stage of myocardial infarction and in the absence of histological signs detectable with conventional staining techniques. However, as highlighted by previous review studies [14,53,54,55,56], the use of different markers (chosen based on their time-correlated specificity) seems appropriate for a correct chronological assessment of acute infarction. Additionally, using a heterogeneous set of markers enhances the specificity of the method: several studies have identified false positive reactions for some markers (such as JunB and Cx43) in cases where the cause of death was determined to be something other than myocardial infarction (e.g., asphyxia) [46,47] although with differing expression patterns. These findings highlight a degree of operator dependence and inter-observer variability, particularly when relying on semi-quantitative evaluation of staining intensity.

Given the evidence that has emerged, a comparative analysis of the principal immunohistochemical markers identified in the review appears to be of particular relevance, with a focus on the diagnostic specificity highlighted by the individual studies.

Based on a focused analysis of the 37 studies included in this review, it was possible to estimate the diagnostic specificity of 14 immunohistochemical markers. Specificity values were extracted directly when reported by the original authors or calculated when sufficient data on marker expression in both infarcted and control samples were available. Markers such as C5b-9, cardiac troponins (T, C, and I), dystrophin, myoglobin, H-FABP, S100A1, and IL-15/MCP-1 consistently showed 100% specificity, as no expression was detected in non-infarcted tissues. In contrast, fibronectin showed a specificity of approximately 87.85%, due to occasional weak staining in control cases. Markers such as ET-1, GDF-15, and dityrosine exhibited lower specificity, ranging from 70 to 90%, while connexin-43 (dephosphorylated) and JunB showed positive expression even in non-cardiac causes of death (e.g., asphyxia), resulting in 0% specificity. Results are summarized in Table 2. This heterogeneity underlines the importance of combining markers with both high sensitivity and specificity in diagnostic panels.

The timing of myocardial infarction represents a crucial issue in forensic pathology. Over the years, various techniques have been employed to enable forensic pathologists to estimate with increasing accuracy the time of onset of myocardial infarction. Dating the infarction is particularly important in cases of death potentially linked to alleged medical malpractice.

Historically, traditional histological methods have been widely used. These techniques have proven to be both sensitive and specific in identifying intermediate and late stages of myocardial infarction but have shown limited utility in assessing the early phases. This limitation stems from the fact that traditional histology relies on identifying morphological changes, which are minimal during the early stages of infarction.

This gap has led to the emergence of immunohistochemistry as a promising technique capable of providing additional information on the early phases of myocardial infarction. This systematic review, conducted following PRISMA guidelines, aimed to analyze the most frequently used immunohistochemical markers to present the current state of the art.

The results showed that several markers highlight different aspects of ischemic damage. Among them, fibronectin, the C5b-9 complex, troponins, and dystrophin emerged as the most reliable. For example, fibronectin has proven particularly useful in detecting altered vascular permeability and can be assessed as early as 15–30 min after the ischemic event. Similarly, C5b-9 is a valuable marker for evaluating complement activation, which induces cell lysis through the complement cascade. According to some authors, this marker increases within 40 min of ischemia onset, while others report its progressive increase up to 2 h post-event.

As is well known, troponins are reliable indicators of myocardial necrosis. These proteins, which mediate muscle contraction, begin to rise approximately 30 min after the onset of ischemia. In contrast, dystrophin—indicative of sarcolemmal integrity loss—does not show significant alterations before 24 h.

In addition to these four principal markers, others such as S100A1, IL-15, and H-FABP have also been studied. Although these have received less attention in the literature, they show promising potential for identifying even earlier stages of damage. These markers reflect various pathological changes related to infarction. For example, during myocardial ischemia, calcium depletion occurs at the cellular level. This phenomenon can be investigated through the immunohistochemical assessment of S100A1, a calcium-binding protein that diminishes rapidly. Studies have shown that S100A1 depletion is associated with ventricular dysfunction, beginning as early as 15 min post-infarction in rats and around 30 min in humans.

Another phenomenon associated with myocardial ischemia is local inflammation, which can be explored through IL-15 detection. Additionally, ischemia causes direct damage to cardiomyocytes, resulting in the release of intracellular proteins. Among the first to be released is H-FABP, a protein involved in fatty acid metabolism. Immunohistochemical quantification of H-FABP is therefore useful for infarction dating.

The evaluation of immunohistochemical marker performance is primarily based on their temporal relationship to the ischemic insult. Markers such as troponins, fibronectin, and S100A1, which change as early as 30 min post-insult, are valuable for identifying the hyperacute phase of myocardial infarction. Conversely, C5b-9 is more suitable for time intervals beyond two hours.

Hence, understanding the temporal expression profile of each marker is critical for postmortem investigations. A recent systematic review by Isailă et al. [57] analyzed 15 studies and summarized early post-mortem immunohistochemical markers including complement proteins (e.g., C9, C5b-9), CD59, cardiac troponins (cTnT, cTnC), myoglobin, desmin, fibrinogen, fibronectin, H-FABP, dityrosine, MIF, inflammatory mediators (IL-1β, IL-6, IL-8, IL-15, MCP-1, TNF-α), adhesion molecules (ICAM-1, CD15, CD18), and stress kinases (p-38, JNK). In contrast, our systematic review screened more studies (37) and examines a broader panel of 49 markers: in addition to confirming the relevance of the markers listed by Isailă et al., we discuss further structural, metabolic and oxidative-stress markers (for example, dystrophin, S100A1, endothelin-1, GDF-15, connexin-43/np-Cx43, JunB, HO-1, Nrf2, adropin, irisin, vimentin, thrombomodulin, von Willebrand factor and several cytoskeletal/apoptotic proteins). Importantly, we provide quantitative estimates of diagnostic specificity for 14 markers and explicitly address markers with limited specificity (e.g., JunB, dephosphorylated Cx43), thereby offering a more practice-oriented, multiparametric framework aimed especially at forensic application.

However, several methodological limitations of this review must be acknowledged. Many of the included studies were based on animal models, which poses challenges when extrapolating findings to human autopsy samples. Furthermore, few studies have systematically assessed the effects of autolytic and putrefactive processes on marker expression—factors that are particularly critical in forensic practice. These degradative processes can lead to rapid loss of intracellular markers, potentially resulting in false negatives.

An additional limitation concerns the technical variability across the reviewed studies, such as differences in antibody types, fixation times, and staining protocols, which hinder standardization of results.

Looking ahead, future perspectives include the development of immunohistochemical panels for accurate myocardial infarction dating, applicable to large human autopsy series. Ideally, these findings should be correlated with detailed clinical and circumstantial data. To establish immunohistochemistry as a routine forensic tool, it will be essential to validate standardized protocols, including those suitable for advanced postmortem conditions.

Another potential improvement lies in the use of multiplex panels capable of simultaneously detecting markers with different expression kinetics—early, intermediate, and late—thereby increasing both diagnostic accuracy and temporal resolution. In our review, only Buğra et al. [20] and Shabaiek et al. [49] provided results regarding marker expression in areas adjacent to the infarcted region. The limited amount of available evidence therefore precludes a comprehensive discussion on the distribution of markers in region adjacent to the infarcted area. In this regard, future studies could better highlight the distribution of markers not only in the ischemic area but also in the surrounding regions.

Finally, a further paradigm shift could be achieved by integrating immunohistochemistry with complementary techniques such as molecular biology, proteomics, and miRNA analysis.

## 4. Conclusions

The diagnosis of sudden cardiac death remains a complex challenge in forensic pathology [58] as do other cardiac conditions under investigation that involve inflammatory or infectious mechanisms [59,60,61,62]. Currently, immunohistochemical analysis, given the considerable number of markers tested on both human samples and animal models, is a valuable tool for forensic pathologists. However, considering the results discussed, particularly in terms of sensitivity and specificity, the use of panels comprising multiple antibodies simultaneously is advisable [19]. Moreover, accurate dating of the infarction would provide significant insights in cases of medical liability. Further studies on immunohistochemical markers tested on human samples and correlated with circumstantial information (such as symptom onset, time of death, and post-mortem interval) are necessary for an accurate and definitive chronological assessment of acute myocardial infarction. The correct diagnosis of early myocardial infarction to date cannot be achieved without the analysis of all other anamnestic, circumstantial, and autoptic elements.

## Figures and Tables

**Table 1:** Summary of the research articles included in the review.

	Authors	Year	Sample	Marker	Time of Expression	Type of Expression
1	Aydin et al. [16]	2014	Rats	Adropin	≤1 h	+
2	Bi et al. [17]	2013	Rats	S100A1	≥15 min	−
			Humans		<6 ore-NHS	
3	MartinezDiaz et al. [18]	2005	Humans	Cardiac troponin C (cTnC) and	NHS	+
				cardiac troponin T (cTnT)		+
4	Brinkmann et al. [19]	1993	Humans	C5b-9	NHS	+
				Desmin		−
				Myoglobin		−
				Fibrinogen		+
5	Buğra et al. [20]	2022	Humans	CD59	NHS	+
				Fibronectin	HS	+
				Cathepsin S	HS	−
				Desmin	NHS and HS	−
				cTnT	NHS and HS	−
				Myoglobin	NHS and HS	−
6	Campobasso et al. [21]	2008	Humans	C5b-9	NHS	+
				cTnI	NHS	−
				Myoglobin	NHS	−
				Fibronectin	NHS	+
7	Erel et al. [22]	2014	Humans	Blc-2	NHS	+
				Bax		+
				Fas		+
8	Falk et al. [23]	2020	Rats	GDF-15	≥15 min	+
				ET-1	≥15 min	+
9	Hansen et al. [24]	1999	Humans	cTnI	NHS	−
10	Hashmi et al. [25]	2013	Rats	Dystrophin	≥20 min	−
11	Hiyamizu et al. [26]	2024	Humans	factor erythroid 2-related factor (nrf2)	NHS	+
				Fibronectin	NHS	+
				C5b-9	NHS	+
12	Hu et al. [27]	1996	Humans	Fibronectin	NHS	+
13	Hu et al. [28]	2019	Rats	Fibronectin	≥15 min	+
				Fibrinogen	≥ 3 h	+
				C5	≥15 min	+
				Myoglobin	≥15 min	−
				Actin hhf35	≥15 min	−
				Desmin	≥15 min	−
14	Kawamoto et al. [29]	2013	Humans	npCX-43	NHS	+
				CX-43	NHS	−
15	Jia et al. [30]	2015	Animals	CtnI	0.5–1 h	+
					≥1 h	−
			Humans		HS	−
16	Kondo et al. [31]	2022	Humans	CD31	HS	+
			Rats		≥4 giorni	
17	Kondo et al. [32]	2021	Humans	Thrombomodulin	HS	+
18	Kondo et al. [33]	2021	Humans	Vimentin	HS	+
19	Kondo et al. [34]	2021	Humans	von Willebrand factor	HS	+
20	Kuloglu et al. [35]	2014	Rats	Irsin	≥1 h	−
21	Kuninaka et al. [36]	2021	Humans	HO-1	NHS	+
22	Mayer et al. [37]	2016	Rats	Dithyrosine	≥5 min	+
23	Mayer et al. [38]	2014	Humans	Dithyrosine	≥4 h	+
24	Meng et al. [39]	2006	Rats	H-FABP	≥15 min	−
			Humans		NHS	
25	Mondello et al. [40]	2018	Humans	Dystrophin	NHS	−
				Fibronectin	NHS	+
				C5b-9	NHS	+
26	Mondello et al. [41]	2021	Humans	Dystrophin	NHS	−
				C5b-9	NHS	+
				Fibronectin	NHS	+
				MMP-9	NHS	+
27	Ortmann et al. [42]	2000	Humans	FABP	NHS	−
				cTnC	NHS	−
				cTnT	NHS	−
				Myoglobin	NHS	−
				Desmin	NHS	−
				CD59	HS	+
				C5b-9	NHS	+
				Fibrinogen	NHS	+
				Fibronectin	NHS	+
28	Ouyang et al. [43]	2009	Humans	Desmin	NHS	−
29	PIercecchi-Marti et al. [44]	2001	Humans	C9	NHS	+
30	Piro et al. [45]	2000	Humans	P53	NHS	−
				Bcl-2	NHS	−
				Cpp32	NHS	−
				FAS	NHS	−
				FAS-L	NHS	−
				Bax	NHS	−
31	Sabatasso et al. [46]	2016	Rats	FIbronectin	≥1 h	−
				C5b-9	≥1 h	+
				cTnI	≥1 h	−
				cTnT	≥1 h	−
				JunB	≥15 min	+
				Myoglobin	≥1 h	+
				Cx43	≥15 min	+
32	Sabatasso et al. [47]	2018	Rats	FIbronectin	≤1 h	−
				C5b-9	≤2 h	+
				cTnT	≤1 h	−
				cTnI	≤1 h	+
				JunB	≤30 min	+
				myoglobin	≤1 h	+
				Cx43	≤30 min	+
33	Scholl et al. [48]	2019	Rats	cTnT	≥5 min	−
				cTnI	≥5 min	−
				Dithyrosine	≥5 min	+
				Cx43	≥5 min	+
34	Shabaiek et al. [49]	2016	Humans	H-FABP	NHS	−
35	Turillazzi et al. [50]	2014	Humans	CD-15	≤4–6 h	+
				IL-1β	≤4–6 h	+
				IL-6	≤4–6 h	+
				TNF-α	≤4–6 h	+
				IL-15	≤4–6 h	+
				IL-8	≤4–6 h	+
				MCP-1	≤4–6 h	+
				ICAM-1	≤4–6 h	+
				CD18	≤4–6 h	+
				tryptase	≤4–6 h	+
36	Watanabe et al. [51]	1993	Humans	FAB-P	NHS	−
37	Zhang et al. [52]	1996	Humans	Vinculin	NHS	−
				Desmin	NHS	−
				α-actinin	NHS	−

Table legend: + → positive staining; − → negative staining; NHS → no histological sign of heart attack (presumed time < 6 h); HS → estimated time > 6 h.

**Table 2:** Summary of different markers investigated.

Marker	Specificity	Sample Type	Included Studies
C5b-9 (MAC)	100%	Human	Mondello, 2018, 2021 [40,41]; Sabatasso, 2016 [46]
Dystrophin	100%	Human and animal	Mondello 2018, 2021[40,41]; Hashmi, 2013 [25]
cTnT/cTnC	100%	Human	Martinez-Diaz, 2005 [18]; Sabatasso, 2016 [46]
cTnI	100%	Human	Sabatasso, 2016 [46]; Jia, 2015 [12]
Myoglobin	100%	Human	Sabatasso, 2016 [46]; Meng, 2006 [39]
H-FABP	100%	Animal	Meng, 2006 [39]; Shabaiek, 2016 [49]
S100A1	100%	Animal	Bi, 2013 [17]
IL-15/MCP-1	100%	Human	Turillazzi, 2014 [50]
ET-1	90%	Human	Falk, 2020 [23]
Fibronectin	87.85%	Human	Hu, 1996 [27]; Mondello, 2018, 2021 [40,41]; Sabatasso, 2016 [46]
Dityrosine	80%	Human	Mayer, 2014, 2016 [37,38]
GDF-15	70%	Human	Falk, 2020 [23]
JunB	0%	Human	Sabatasso, 2016, 2018 [46,47]
Cx43	0%	Human	Sabatasso, 2016, 2018 [46,47]
